# Incidence, Clinical Characteristics, and Predictors of Cardiovascular Immune-Related Adverse Events Associated with Immune Checkpoint Inhibitors

**DOI:** 10.1093/oncolo/oyac056

**Published:** 2022-03-28

**Authors:** Tsuyoshi Isawa, Yukihiro Toi, Shunichi Sugawara, Masataka Taguri, Shigeru Toyoda

**Affiliations:** 1 Department of Cardiology, Sendai Kousei Hospital, Sendai, Japan; 2 Department of Pulmonary Medicine, Sendai Kousei Hospital, Sendai, Japan; 3 Department of Data Science, Yokohama City University School of Data Science, Yokohama, Japan; 4 Department of Cardiovascular Medicine, Dokkyo Medical University, Mibu, Japan

**Keywords:** cardiotoxicity, oncology, immune checkpoint inhibitors, immunotherapy, lung neoplasms

## Abstract

**Background:**

Cardiovascular immune-related adverse events (CV–irAEs) associated with immune checkpoint inhibitors (ICIs) may have been underreported given that most previous reports were retrospective. We aimed to evaluate the incidence, clinical characteristics, and predictors of CV–irAEs and determine the feasibility of serial cardiac monitoring using a combination of B-type natriuretic peptide, cardiac troponin T, and electrocardiogram for the prediction of future symptomatic (grade ≥2) CV–irAEs.

**Materials and Methods:**

This was a prospective observational study that included 129 consecutive patients with non–small-cell lung cancer who received ICI monotherapy at a single center. Serial cardiac monitoring was performed during ICI monotherapy.

**Results:**

A total of 35 (27%) patients developed any grade ≥1 CV–irAEs with a median time of onset of 72 (interquartile range 44-216) days after ICI treatment initiation. Multivariate Fine–Gray regression analysis showed that prior acute coronary syndrome (adjusted hazard ratio [HR] 3.15 (95% [CI], 2.03-4.91), prior heart failure hospitalization (adjusted HR 1.65 [95% CI, 1.17-2.33]), and achievement of disease control (adjusted HR 1.91, [95% CI, 1.16-3.14]) were significantly associated with grade ≥1 CV–irAEs. Serial cardiac monitoring revealed that patients with preceding grade 1 CV–irAEs were associated with a significantly higher risk of onset of grade ≥2 CV–irAEs compared with those without preceding grade 1 CV–irAEs (HR: 6.17 [95% CI, 2.97-12.83]).

**Conclusion:**

CV–irAEs were more common than previously recognized and have several predictors. Moreover, serial cardiac monitoring may be feasible for the prediction of future grade ≥2 CV–irAEs.

## Teaser

This article evaluates the incidence, clinical characteristics, and predictors of cardiovascular immune-related adverse events (CV-irAEs), focusing on the feasibility of serial cardiac monitoring using a combination of B-type natriuretic peptide, cardiac troponin T, and electrocardiogram for the prediction of future symptomatic (grade ≥2) CV-irAEs

**Implications for Practice**

This single-center prospective observational study included 129 consecutive patients with nonsmall–cell lung cancer who received ICI monotherapy, and revealed that preceding grade 1 CV-irAEs conferred a significantly higher risk of potential grade ≥2 CV-irAEs. Therefore, serial cardiac monitoring may be feasible for the prediction of future grade ≥2 CV-irAEs.

## Introduction

Immune-related adverse events (irAEs) associated with immune checkpoint inhibitors (ICIs) are side effects related to enhanced immune system activity caused by ICIs, which can affect multiple organs, including the skin, gastrointestinal tract, liver, lungs, endocrine system, renal system, musculoskeletal system, and cardiovascular system.^1^ The recently published clinical practice guideline of the American Society of Clinical Oncology (ASCO) defined irAEs and their grading, and indicated that cardiovascular immune-related adverse events (CV–irAEs) include not only myocarditis but also pericarditis, arrhythmia, heart failure, and vasculitis.^2^ Moreover, CV–irAEs defined by the ASCO guidelines included abnormal cardiac biomarker results, such as cardiac troponin and abnormal electrocardiogram (ECG) findings, without any concurrent cardiovascular symptoms (asymptomatic CV–irAEs, grade 1). However, considering that most previous reports on CV–irAEs were retrospective and focused mainly on symptomatic ICI–related acute myocarditis, thus possibly overlooking asymptomatic cases, CV–irAEs may have been underdiagnosed and underreported.^3,4^ As such, the actual incidence of CV–irAEs, including asymptomatic cases, remains unclear. Therefore, a pilot registry was established to prospectively evaluate the incidence, clinical characteristics, and predictors of CV–irAEs, including asymptomatic ones, in real-world patients. Furthermore, this registry aimed to determine the feasibility of serial monitoring (prospective screening) using a combination of B-type natriuretic peptide (BNP), cardiac troponin T, and ECG for the early detection of future symptomatic CV–irAEs.

## Materials and Methods

### Patient Selection

This single-center prospective observational study included patients with non–small-cell lung cancer (NSCLC) who underwent ICI monotherapy. A total of 129 consecutive patients with NSCLC who received ICI monotherapy at the Sendai Kousei Hospital between May 2018 and December 2019 were enrolled. There were no patients who failed to receive serial cardiac monitoring or refused to participate during the study period. Patients underwent the following ICI monotherapy every 2-3 weeks until documented disease progression, intolerable toxicity levels, or termination by the physician: nivolumab (3 mg/kg or 240 mg every 2 weeks), pembrolizumab (200 mg every 3 weeks), atezolizumab (1200 mg every 3 weeks), and durvalumab (10 mg/kg every 2 weeks). No patients received concurrent chemotherapy or radiotherapy during serial cardiac monitoring. The study protocol was approved by the Institutional Review Board of the Sendai Kousei Hospital (approval number, 30-8; approval date, May 16, 2018), and all patients provided written informed consent prior to their enrollment. All procedures performed in this study were in accordance with the ethical standards of the institutional research committee and the 1964 Declaration of Helsinki and its later amendments or comparable ethical standards. The study was registered at the University Hospital Medical Information Network Clinical Trials Registry, as accepted by the International Committee of Medical Journal Editors (No. UMIN000032729).

### Endpoint Definition

The baseline laboratory data, including BNP and cardiac troponin T levels (qualitative measurement), were collected before ICI treatment. The cardiac troponin T level was measured using a rapid bedside assay with a cutoff value of 0.1 ng/mL (TropT, Roche Diagnostics, Mannheim, Germany). The ECG and transthoracic echocardiography data before ICI treatment were also obtained. Serial cardiac monitoring included BNP, cardiac troponin T, and ECG, all of which were performed at baseline (before ICI treatment) and once every 4-6 weeks depending on the timing of ICI administration. Moreover, additional measurements of BNP, cardiac troponin T, or ECG were conducted other than the scheduled ones at the discretion of the attending pulmonary physicians. Each ECG finding was reviewed by both an attending pulmonary physician and a dedicated cardiologist. When BNP elevation (≥200 pg/mL), cardiac troponin T conversion, or new-onset morphological ECG abnormalities were documented during ICI treatment, cardiology consultation was always considered and, if necessary, transthoracic echocardiography and cardiac catheterization, including coronary angiography and endomyocardial biopsy, were performed. On the basis of the definition by the ASCO (Supplementary Table S1), grade 1 CV–irAEs (asymptomatic CV–irAEs) were characterized as a composite of BNP elevation ≥200 pg/mL, cardiac troponin T conversion, or new-onset morphological ECG abnormalities without any cardiovascular symptoms. Grades 2 and 3 CV–irAEs were characterized as a composite of BNP elevation to ≥200 pg/mL, cardiac troponin T conversion, or new-onset morphological ECG abnormalities with mild (grade 2) or more severe (grade 3) cardiovascular symptoms. Furthermore, grade 4 CV–irAEs were characterized as a composite of BNP elevation to ≥200 pg/mL, cardiac troponin T conversion, or new-onset morphological ECG abnormalities with life-threatening conditions or severe conditions requiring immediate IV medication or intervention. The difference between grades 2 and 3 events as defined by the ASCO guidelines is ambiguous because both definitions are based on subjective judgment. Therefore, to avoid ambiguity, we did not distinguish between grade 2 and 3 CV–irAEs. Instead, we merged “grade 2,” “grade 3,” and “grade 4” events into the “grade ≥2” events category. BNP elevation ≥200 pg/mL was defined as the BNP level that is lower than 200 pg/mL at baseline, which subsequently increased to 200 pg/mL or more after ICI treatment initiation. This was intended to exclude cancer-related BNP elevation as much as possible because plasma BNP levels have been reported to be elevated, although usually not exceeding 200 pg/mL, due to cancer-related inflammation in advanced cancer patients.^5^ Cardiac troponin T conversion was defined as a negative troponin T-test result at baseline that subsequently turned positive at least once after ICI treatment initiation. New-onset morphological ECG abnormalities included any morphological changes with respect to the baseline ECG results. Patients on ICI treatment with concurrent diseases that could potentially explain the laboratory abnormalities, including myocardial oxygen supply/demand mismatch in the setting of sepsis, anemia, and hypoxia, and patients with concurrent diseases that could cause false-positive troponin T results (eg, rhabdomyolysis) were not counted as developing CV–irAEs. Moreover, patients with BNP levels elevated to ≥200 pg/mL or positive cardiac troponin T-test results prior to beginning ICI treatment were also not counted as developing CV–irAEs, even if they had either positive troponin T-test results or BNP levels elevated to ≥200 pg/mL during ICI treatment. Additionally, if the patients on ICI treatment developed the same ECG abnormalities as those already documented before ICI treatment, eg, paroxysmal atrial fibrillation and negative T-wave, they were not counted as developing CV–irAEs. Symptomatic (grade ≥2) CV–irAEs included (i) biopsy-proven acute myocarditis; (ii) acute decompensated heart failure, the cardiogenic shock of unknown etiology, or heart failure deterioration in at least one New York Heart Association functional class; (iii) life-threatening arrhythmias, including advanced or complete atrioventricular block and ventricular tachycardia or fibrillation; (iv) non-lethal arrhythmias causing cardiovascular symptoms, including atrial fibrillation or flutter; and (v) cardiac death, new-onset acute coronary syndromes, or any coronary revascularization procedure. irAEs, except for CV–irAEs, were assessed by an attending pulmonary physician and a dedicated nurse specialist every 2-3 weeks throughout the course of ICI treatment and were graded according to the ASCO definition.^2^

### Statistical Analysis

Continuous variables were presented as medians [interquartile ranges (IQRs)], while categorical variables were presented as frequencies (percentages). Univariate and multivariate Fine and Gray regression models for grade ≥1 CV–irAEs were performed to evaluate the associations among baseline clinical, laboratory, and treatment characteristics with grade ≥1 CV–irAEs, considering non-cardiovascular death as a competing event. Predictors of grade ≥1 CV–irAEs were initially screened using a univariate Fine and Gray regression model. The level of significance for the univariate screening regressions was set at a *P*-value of <.05. Thereafter, a multivariate Fine and Gray regression model using variables with a *P*-value of <.05 in the univariate analysis was established to estimate hazard ratios (HRs) and 95% CIs. Similarly, univariate and multivariate Fine and Gray regression analyses were also performed to assess the factors related to grade ≥2 CV–irAEs. All statistical analyses were performed using the EZR software (version 1.53; Saitama Medical Center, Jichi Medical University; http://www.jichi.ac.jp/saitama-sct/SaitamaHP.files/statmedEN.html), a graphical user interface for R (The R Foundation for Statistical Computing, Vienna, Austria), with a two-sided *P*-value of <.05 indicating statistical significance.

## Results

Among the 129 patients analyzed in this study, 47 (36%) received nivolumab monotherapy, 46 (36%) received pembrolizumab, 17 (13%) received atezolizumab, and 19 (15%) received durvalumab throughout the study period (Supplementary Table S2).

**Figure 1.**
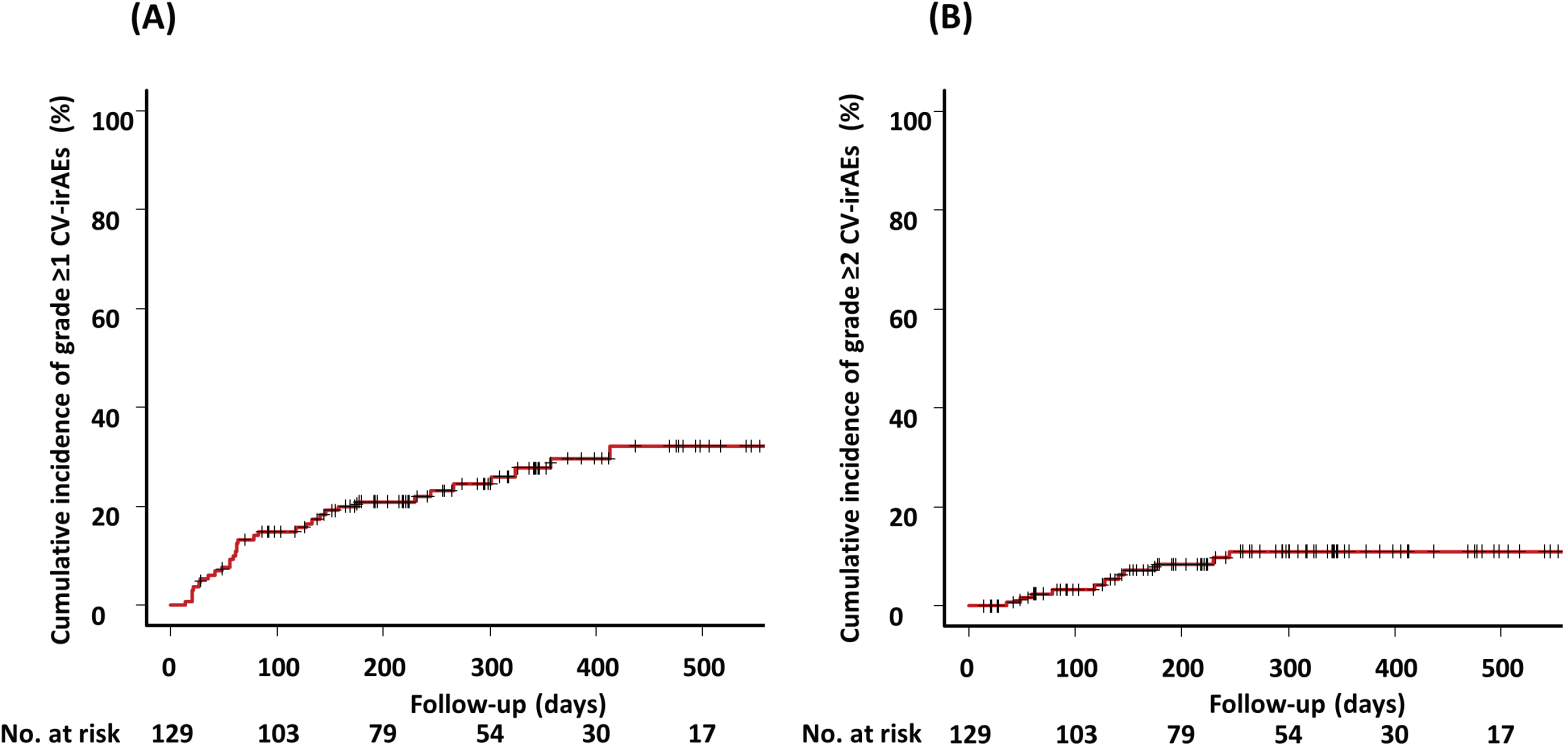
Cumulative incidence of CV–irAEs. (**A**) Grade ≥1 CV–irAEs. (**B**) Grade ≥2 CV–irAEs. Cumulative incidence of CV–irAEs was estimated, considering non–cardiovascular death as a competing event. CV–irAEs, cardiovascular immune-related adverse events.

The observed clinical events and abnormal laboratory findings after ICI treatment initiation among the 129 patients analyzed herein are presented in Table 1. BNP elevation ≥200 pg/mL only after ICI treatment initiation, cardiac troponin T conversion, and any new-onset morphological ECG abnormalities were observed in 12%, 10%, and 11% of the patients, respectively. A total of 41 patients had BNP elevation (≥200 pg/mL), cardiac troponin T conversion, or new-onset morphological ECG abnormalities during ICI treatment. Among them, the six patients who had concurrent diseases that could potentially cause abnormal increases in BNP level, cardiac troponin T conversion, or new-onset ECG abnormalities were identified during ICI treatment and were excluded from the grade ≥1 CV–irAE group. Of the six patients excluded, two patients with cardiac troponin T conversion experienced sepsis and rhabdomyolysis, respectively, three patients with paroxysmal atrial fibrillation experienced hypoxia secondary to advanced lung cancer, and one patient with BNP elevation developed severe anemia (possible anemia-induced heart failure). Consequently, 35 (27%) patients developed any grade ≥1 CV–irAEs with a median time of onset of 72 (IQR 44-216) days after ICI treatment initiation over a median follow-up duration of 255 days (IQR 134-386) (Fig. 1A). Additionally, 13 (10%) patients developed any grade ≥2 CV–irAEs with a median time of onset of 141 (IQR 69-234) days after ICI treatment initiation (Fig. 1B). Among patients with CV–irAEs, no cardiac death occurred during the follow-up period. Table 2 reveals the univariate associations among the baseline clinical, laboratory, and treatment characteristics between NSCLC patients with and without grade ≥1 CV–irAEs who underwent ICI treatment based on Fine and Gray competing risk analysis. Accordingly, significantly more patients with grade ≥1 CV–irAEs had prior histories of acute coronary syndrome and heart failure hospitalization compared to patients without CV–irAEs (HR 1.91 [95% CI, 1.52-2.41], *P* < .001 and HR 1.91 [95% CI, 1.52-2.41], *P* < .001, respectively). Moreover, significantly more patients with grade ≥1 CV-irAEs had spirometry-defined chronic obstructive pulmonary disease or emphysema on computed tomography (CT), performance status ≥2, achievement of disease control (complete response, partial response, or stable disease), and negative T-wave before ICI treatment compared with patients without CV-irAEs (HR 1.56 [95% CI, 1.02-2.38], *P* = .039; HR 1.91 [95% CI, 1.52-2.41], *P* < .001; HR 2.04 [95% CI, 1.28-3.25], *P* = .003; and HR 2.00 [95% CI, 1.56-2.57], *P* < .001, respectively). The results of the multivariate Fine and Gray regression model for grade ≥1 CV–irAEs in patients with NSCLC who underwent ICI treatment, considering non-cardiovascular death as a competing event, are shown in Table 3. Negative T-wave, spirometry-defined chronic obstructive pulmonary disease or emphysema on CT, and performance status ≥2 were not independently associated with grade ≥1 CV–irAEs. By contrast, prior acute coronary syndrome (adjusted HR 3.15 [95% CI, 2.03-4.91], *P* < .001), prior heart failure hospitalization (adjusted HR 1.65 [95% CI, 1.17-2.33], *P* = .004), and achievement of disease control (adjusted HR 1.91 [95% CI, 1.16-3.14], *P* = .011) were significantly associated with grade ≥1 CV–irAEs. Additionally, the univariate and multivariate Fine and Gray regression models for grade ≥2 CV–irAEs in patients with NSCLC who underwent ICI treatment, considering non-cardiovascular death as a competing event, showed that performance status (PS) ≥2 (adjusted HR 84.62, [95% CI, 18.43-388.40], *P* < .001) and achievement of stable disease (adjusted HR 3.63 [95% CI, 1.22-10.77], *P* = 0.020) were significantly associated with grade ≥2 CV–irAEs (Supplementary Tables S3 and S4).

**Table 1.** Observed clinical events and abnormal laboratory findings after ICI treatment initiation among the 129 patients analyzed in this study.

Clinical events and abnormal laboratory findings	*n* (%)	Days to onset, median (IQR), days
BNP elevation ≥200 pg/mL only after ICI treatment initiation	15 (12)	128 (59-302)
Cardiac troponin T conversion	13 (10)	56 (24-126)
**New-onset morphological ECG abnormalities** ^a^	14 (11)	124 (53-284)
Negative T-wave	3 (2)	147 (63-158)
ST-T change	2 (2)	190 (22-358)
Paroxysmal atrial fibrillation	7 (5)	175 (36-238)
Trifascicular block	1 (1)	391 (n/a)
Life–threatening arrhythmias^b^	2 (2)	207 (92-321)
**Biopsy-proven acute myocarditis**	1 (1)	419 (n/a)
**ADHF or heart failure deterioration in at least one NYHA functional class**	6 (5)	128 (58-213)
**Pericarditis**	0 (0)	n/a
**Vasculitis**	0 (0)	n/a
**CV–irAEs defined by the ASCO**		
Grade ≥1^c^	35 (27)	72 (44-216)
Grade 1^d^	22 (17)	62 (26-185)
Grade ≥2	13 (10)	141 (69-234)
With preceding grade 1 CV–irAEs	6 (5)	123 (45-247)
Without preceding grade 1 CV–irAEs	7 (5)	161 (72-318)
Grade 4^e^	3 (2)	321 (207-370)

The same patient developed both ventricular tachycardia and paroxysmal atrial fibrillation during the follow-up period.

Two patients developed life–threatening arrhythmia: one patient developed complete atrioventricular block and the other patient developed ventricular tachycardia.

Patients with grade ≥1 CV–irAEs included both patients with grade 1 events alone (22 patients) and patients with grade ≥2 events (13 patients) during follow-up.

Patients who developed grade 1 events prior to grade ≥2 events were counted only as having grade ≥2 events.

Three patients had grade 4 CV–irAEs: one with acute myocarditis, one with ventricular tachycardia, and one with complete atrioventricular block requiring pacemaker implantation, respectively.

Abbreviations: ADHF: acute decompensated heart failure; ASCO: American Society of Clinical Oncology; BNP: B-type natriuretic peptide; CV–irAEs: cardiovascular immune-related adverse events; ECG: electrocardiogram; ICI: immune checkpoint inhibitor; IQR: interquartile range; n/a: not applicable; NYHA: New York Heart Association.

**Table 2.** Univariate associations among baseline clinical, laboratory, and treatment characteristics and grade ≥1 CV–irAEs in patients with NSCLC undergoing ICI treatment based on Fine and Gray competing risk analysis, considering non–cardiovascular death as a competing event.

Variables	With CV–irAEs (grade≥1) *n* = 35	Without CV–irAEs *n* = 94	HR^a^ (95% CI)	*P-*value
Age, median (IQR), years	71 (65-78)	71 (64-77)	0.99 (1.00-1.02)	.67
Sex (male), *n* (%)	28 (80)	72 (77)	0.98 (0.56-1.72)	.95
Height, median (IQR), m	163 (156-168)	162 (156-168)	1.01 (0.98-1.04)	.54
Body weight, median (IQR), kg	55.0 (46.0-64.0)	55.5 (47.0-67.0)	1.01 (0.99-1.03)	.26
Body mass index, median (IQR), kg/m^2^	20.0 (18.4-24.7)	21.7 (18.4-24.5)	1.03 (0.97-1.08)	.36
Diabetes, *n* (%)	5 (14)	25 (27)	0.62 (0.30-1.30)	.20
Hypertension, *n* (%)	18 (51)	54 (57)	0.83 (0.53-1.30)	.42
Active or ex-smoking, *n* (%)	31 (89)	82 (87)	1.07 (0.51-2.23)	.86
Prior stroke, *n* (%)	0 (0)	4 (4)	n/a	n/a
Prior ACS, *n* (%)	1 (3)	2 (2)	1.91 (1.52-2.41)	<.001
Prior heart failure hospitalization, *n* (%)	1 (3)	1 (1)	1.91 (1.52-2.41)	<.001
Spirometry-defined COPD or emphysema on CT, *n* (%)	11 (31)	24 (26)	1.56 (1.02-2.38)	.039
ECOG PS^b^, *n* (%)				
0	27 (77)	68 (72)	1.47 (0.81-2.64)	.20
1	7 (20)	25 (27)	0.62 (0.33-1.17)	.14
≥2	1 (3)	1 (1)	1.91 (1.52-2.41)	<.001
Cancer stage^c^, *n* (%)				
II	8 (23)	28 (30)	1.33 (0.82-2.16)	.24
IV	27 (77)	65 (69)	0.75 (0.46-1.21)	.24
Pathological subtype, *n* (%)				
SCC	18 (51)	43 (46)	1.06 (0.67-1.67)	.81
Nonsquamous NSCLC	15 (49)	51 (54)		
Prior use of anthracyclines, *n* (%)	0 (0)	0 (0)	n/a	n/a
Prior use of VEGF inhibitors^d^, *n* (%)	4 (11)	10 (11)	0.72 (0.33-1.60)	.42
Prior radiation, *n* (%)	7 (20)	38 (40)	0.67 (0.36-1.25)	.20
PD-L1 expression, *n* (%)				
TPS ≥50%	8 (24)	17 (20)	1.46 (0.92-2.30)	.11
Nivolumab, *n* (%)	16 (46)	31 (33)	0.89 (0.57-1.41)	.63
Pembrolizumab, *n* (%)	14 (40)	32 (34)	1.43 (0.92-2.21)	.11
Atezolizumab, *n* (%)	4 (11)	13 (14)	0.65 (0.29-1.46)	.29
Durvalumab, *n* (%)	1 (3)	18 (19)	0.94 (0.23-3.84)	.93
Best response, *n* (%)				
Complete response	0 (0)	1 (1)	n/a	n/a
Partial response	8 (23)	26 (28)	1.66 (1.10-2.51)	.016
Stable disease	13 (37)	20 (21)	1.58 (1.04-2.40)	.034
Progressive disease	14 (40)	47 (50)	0.49 (0.31-0.78)	.003
Objective response^e^	8 (23)	27 (29)	1.66 (1.10-2.51)	.016
Disease control^f^	21 (60)	47 (50)	2.04 (1.28-3.25)	.003
Pre-ICI treatment white blood cells (×10^3^), median (IQR), per µL	6.2 (5.1-7.2)	6.2 (4.3-7.5)	1.0 (n/a)	.33
Pre-ICI treatment hemoglobin, median (IQR), g/dL	12.4 (11.4-13.3)	12.1 (10.8-13.0)	1.14(1.00-1.29)	.05
Pre-ICI treatment eGFR, median (IQR), mL/min/1.73 m^2^	69.0 (56.7-79.9)	75.9 (60.7-87.4)	1.00 (0.99-1.01)	.75
Pre-ICI treatment BNP, median (IQR), pg/mL	42.1 (24.4-58.7)	26.7 (13.5-62.6)	1.0 (n/a)	.85
Clinically relevant BNP elevation (≥200 pg/mL) at the start of ICI treatment^g^, *n* (%)	0 (0)	3 (3)	n/a	n/a
Pre-ICI treatment echocardiogram				
LVEF, median (IQR), %	76 (69-78)	70 (66-75)	1.01 (0.98-1.03)	.73
LVEd, median (IQR), mm	43 (42-45)	44 (41-48)	0.99 (0.94-1.04)	.62
LVEs, median (IQR), mm	25 (23-28)	27 (24-30)	0.99 (0.93-1.05)	.67
Clinically relevant LVEF dysfunction (<50%) at the start of ICI treatment^g^, *n* (%)	0 (0)	2 (2)	n/a	n/a
Cardiac troponin T prior to ICI use, *n* (%)				
Positive	0 (0)	2 (2)	n/a	n/a
Pre-ICI treatment ECG				
Persistent/paroxysmal atrial fibrillation, *n* (%)	2 (6)	15 (16)	0.94 (0.34-2.57)	.90
ST-T changes, *n* (%)	2 (6)	2 (2)	1.27 (0.55-2.93)	.57
Negative T-wave, *n* (%)	4 (11)	1 (1)	2.00 (1.56-2.57)	<.001
Heart rate at rest, median (IQR), per min	76 (67-89)	80 (68-87)	1.00 (0.98-1.01)	.83
QRS width, median (IQR), ms	100 (80-120)	90 (80-100)	1.01 (1.00-1.02)	.07
Other organ systems affected by irAEs, *n* (%)				
Skin reaction	6 (17)	15 (16)	1.16 (0.66-2.04)	.61
Myositis/peripheral neuropathy	1 (3)	2 (2)	0.62 (0.12-3.11)	.56
Hypothyroidism/hyperthyroidism	5 (14)	8 (9)	1.41 (0.83-2.39)	.21
Pneumonitis	5 (14)	17 (18)	1.41 (0.83-2.39)	.21
Hepatitis	3 (9)	8 (9)	0.68 (0.27- 1.71)	.41
Diarrhea	1 (3)	1 (1)	0.94 (0.23-3.84)	.93
Nephritis	1 (3)	1 (1)	1.91 (1.52-2.41)	<.001
Adrenal insufficiency	2 (6)	4 (4)	1.27 (0.55-2.93)	.57
Any irAEs other than CV–irAEs	18 (51)	43 (46)	1.06 (0.67-1.67)	.81

‘Without CV–irAEs’ was considered a reference category.

Scores range from 0 to 4, with higher numbers indicating greater disability.

Among the 129 patients analyzed, one was diagnosed with at least stage II or higher NSCLC and was, therefore, excluded from either stage III or stage IV.

VEGF inhibitors included bevacizumab and ramucirumab.

Achieving complete or partial response.

Achieving complete response, partial response, or stable disease.

The term, “clinically relevant”, was defined as either BNP elevation (≥200 pg/mL) or LVEF dysfunction (<50%) originating from symptomatic (New York Heart Association class II or greater) chronic heart failure at the start of ICI treatment.

Abbreviations: ACEI: angiotensin-converting enzyme inhibitor; ACS: acute coronary syndrome; ARB: angiotensin II receptor blocker; BNP: B-type natriuretic peptide; CI: confidence interval; CT: computed tomography; COPD: chronic obstructive pulmonary disease; CV–irAEs: cardiovascular immune-related adverse events; ECOG PS: Eastern Cooperative Oncology Group Performance Status Scale; ECG: electrocardiogram; eGFR: estimated glomerular filtration rate; HR: hazard ratio; ICI: immune checkpoint inhibitor; IQR: interquartile range; LVEd: left ventricular end-diastolic dimension; LVEF: left ventricular ejection fraction; LVEs: left ventricular end-systolic dimension; n/a: not applicable; NSCLC: non–small-cell lung cancer; PD-L1: programmed death-ligand 1; SCC: squamous cell carcinoma; TPS: tumor proportion score; VEGF: vascular endothelial growth factor.

**Table 3.** Univariate and multivariate Fine and Gray regression models for grade ≥1 CV–irAEs in patients with NSCLC undergoing ICI treatment, considering non–cardiovascular death as a competing event.

Variables	Univariate		Multivariate^a^	
	HR (95% CI)	*P-*value	AHR (95% CI)	*P-*value
Prior ACS	1.91 (1.52-2.41)	<.001	3.15 (2.03-4.91)	<.001
Prior heart failure hospitalization	1.91 (1.52-2.41)	<.001	1.65 (1.17-2.33)	.004
Negative T-wave before ICI treatment	2.00 (1.56-2.57)	<.001	1.65 (1.00-2.73)	.050
Spirometry-defined COPD or emphysema on CT	1.56 (1.02-2.38)	.039	1.48 (0.96-2.27)	.077
ECOG PS ≥2	1.91 (1.52-2.41)	<.001	1.00 (0.61-1.63)	1.000
Achievement of disease control	2.04 (1.28-3.25)	.003	1.91 (1.16-3.14)	.011

Adjusted for prior ACS, prior heart failure hospitalization, negative T-wave before ICI treatment, spirometry-defined COPD or emphysema on CT, ECOG PS ≥ 2, and achievement of disease control. ICI–related nephritis was not included in the multivariate regression model because it could cause a false-positive troponin T result regardless of the presence of cardiovascular events. Disease control was chosen as the representative best response categorization variable. There was no multicollinearity among prior ACS, prior heart failure hospitalization, and negative T-wave before ICI treatment.

ACS, acute coronary syndrome; AHR, adjusted hazard ratio; CI, confidence interval; COPD, chronic obstructive pulmonary disease; CT, computed tomography; CV–irAEs, cardiovascular immune-related adverse events; ECOG PS, Eastern Cooperative Oncology Group Performance Status Scale; HR, hazard ratio; ICI, immune checkpoint inhibitor; NSCLC, non-small-cell lung cancer.

**Figure 2.**
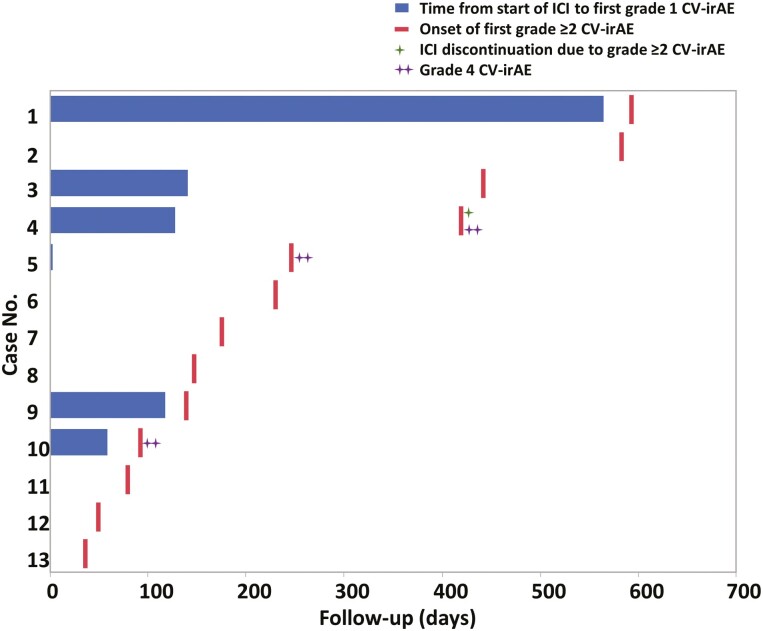
Cumulative incidence of grade ≥2 CV–irAEs stratified by preceding grade 1 CV–irAEs. Cumulative incidence of grade ≥2 CV–irAEs was estimated, considering non–cardiovascular death as a competing event. CV–irAEs, cardiovascular immune-related adverse events.

**Figure 3.**
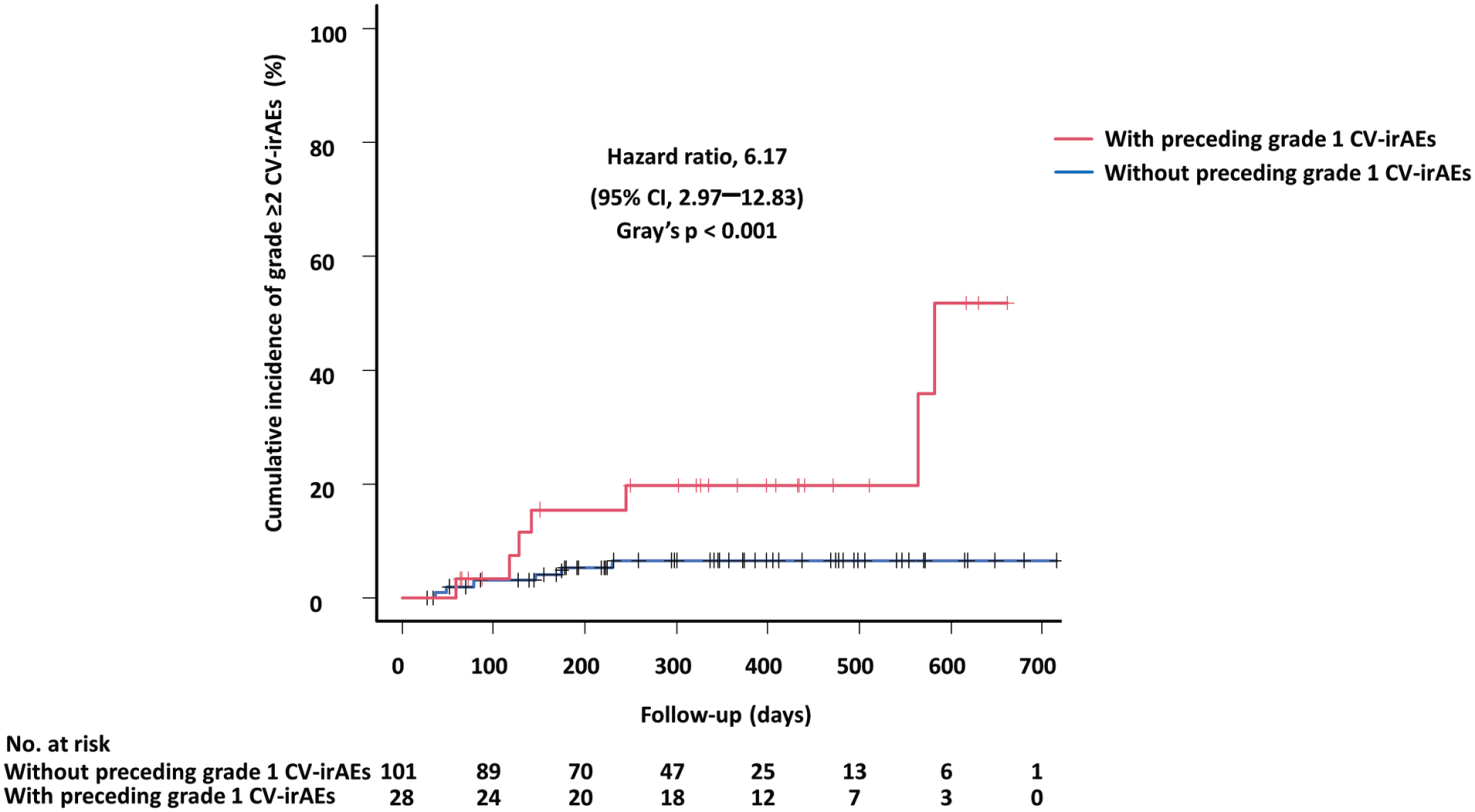
Horizontal histogram depicting total elapsed days from ICI treatment initiation for non–small-cell lung cancer to CV–irAEs in each of the 13 patients who developed grade ≥2 CV–irAEs. The median time interval between the onset of preceding grade 1 and subsequent grade ≥2 CV–irAE was 31 days (interquartile range 29-279 days). Abbreviations: CV–irAEs: cardiovascular immune-related adverse events; ICI: immune checkpoint inhibitor.

Serial cardiac monitoring revealed that patients with preceding grade ≥1 CV–irAEs had a significantly higher risk of subsequent onset of grade ≥2 CV–irAEs compared to patients without preceding grade 1 CV–irAEs (HR: 6.17 [95% CI, 2.97-12.83], *P* < .001; Fig. 2). Among the 13 patients who developed grade ≥2 CV–irAEs, 6 patients (46%) experienced preceding grade 1 CV–irAEs before the onset of grade ≥2 CV–irAEs. Patients with grade ≥2 CV–irAEs as their first grade ≥2 CV–irAEs after ICI treatment initiation included 6 patients (46%) with acute decompensated heart failure, 5 patients (38%) with paroxysmal atrial fibrillation, 1 patient (8%) with biopsy-proven acute myocarditis, and 1 patient (8%) with complete atrioventricular block. None of these patients required discontinuation of ICI due to grade ≥2 CV–irAEs except for 1 patient with biopsy-proven ICI–related acute myocarditis (Fig. 3 and Table 4).

**Table 4.** Clinical characteristics, investigations, and treatments of 13 patients with grade ≥2 CV–irAEs.

Case no.	Age Sex	Stage	ECOG PS	Histological type	Type of ICIs	Pre-ICI LVEF, %	ECG Pre-ICI/during ICI	G 1 days to onset	G 1	Resolution of G 1	G ≥2 days to onset
1	80M	IV	0	Adeno	P	73	Normal/normal	565	BNP elevation	No	593
2	62M	IV	0	Sq	N	82	Normal/Af				583
3	71M	IV	0	Adeno	N	82	Normal/normal	141	BNP elevation	No	442
4	75F	IV	1	Sq	N	58	Normal/normal	128	BNP elevation	No	419/419
5	69M	III	0	Sq	N	70	Normal/Af	3	BNP elevation	No	246/321
6	79M	IV	0	Sq	P	59	Negative T-wave/Af				230/231
7	62M	IV	0	Sq	N	63	Normal/Af				175
8	71F	IV	0	Sq	N	57	Normal/negative T-wave				147
9	81M	III	0	Sq	P	83	Normal/normal	118	Troponin T conversion	No	139
10	79M	IV	3	Adeno	N	77	Negative T-wave/CAVB	59	BNP elevation	No	92
11	74M	IV	1	Adeno	N	78	Normal/Af				79
12	65M	III	0	Sq	A	77	Normal/Af				49
13	66M	IV	0	Adeno	P	75	Normal/Af				36

Case no.	G ≥2	Intervals^a^, days	TnT during ICI treatment^b^ Pre-ICI/peak BNP level (pg/mL)	Advanced diagnostics: CAG/CMR/EMBx	Management	ICI discontinuation^c^					
1	ADHF	28	TnT: negative BNP: 134/689	None	Diuretics Fluid restriction	No					
2	Paf		TnT: negative BNP: 34/92	None	Follow-up^d^	No					
3	ADHF	301	TnT: negative BNP: 90/880	None	Diuretics	No					
4	ADHF/acute myocarditis^e^	291	TnT: negative BNP: 123/490	CAG and EMBx	Diuretics β-blocker	Yes					
5	Paf/VT^e^	243	TnT: negative BNP: 70/713	None	β-blocker	No					
6	ADHF/Paf	168	TnT: negative BNP: 189/808	None	Diuretics	No					
7	Paf		TnT: negative BNP: 12/129	None	Follow-up^d^	No					
8	ADHF		TnT: negative BNP: 38/668	CAG and EMBx	Bed rest Fluid restriction	No					
9	ADHF	21	TnT: positive BNP: 15/56	None	Diuretics	No					
10	CAVB^e^	33	TnT: negative BNP: 54/249	CAG and EMBx	PMI	No					
11	Paf		TnT: negative BNP: 4/124	None	Electrical cardioversion	No					
12	Paf		TnT: negative BNP: 43/107	None	Pharmacological cardioversion	No					
13	Paf		TnT: negative BNP: 25/ 99	None	β-blocker	No					

Time intervals between the onset of the first grade 1 event and the first development of grade ≥2 event.

A cutoff of 0.1 ng/mL (TropT, Roche Diagnostics, Mannheim, Germany).

ICI treatment discontinuation exclusively due to cardiovascular immune-related adverse events.

The symptoms of atrial fibrillation were very mild, and therefore clinical follow-up was chosen without rate or rhythm control.

Grade 4 CV–irAE.

BNP elevation was defined as a BNP level below 200 pg/mL at baseline that subsequently increased to 200 pg/mL or more after ICI treatment initiation. Cardiac troponin T conversion was defined as a negative troponin T-test result at baseline that subsequently turned positive at least once after ICI treatment initiation.

Abbreviations: A: atezolizumab; Adeno: adenocarcinoma; ADHF: acute decompensated heart failure; Af: atrial fibrillation; BNP: B-type natriuretic peptide; CAG: coronary angiography; CAVB: complete atrioventricular block; CMR: cardiac magnetic resonance imaging; CV–irAEs: cardiovascular immune-related adverse events; ECG: electrocardiography; ECOG PS: Eastern Cooperative Oncology Group Performance Status Scale; EMBx: endomyocardial biopsy; G 1: grade 1 cardiovascular immune-related adverse events; G ≥2: grade ≥2 cardiovascular immune-related adverse events; ICI:immune checkpoint inhibitor; LVEF: left ventricular ejection fraction; N: nivolumab; P: pembrolizumab; Paf: paroxysmal atrial fibrillation; PMI: permanent pacemaker implantation; Sq: squamous cell carcinoma; TnT: troponin T; TTE: transthoracic echocardiography; VT: ventricular tachycardia.

## Discussion

To the best of our knowledge, this is the first prospective observational study that evaluated the incidence, clinical characteristics, and predictors of CV–irAEs in patients with NSCLC receiving ICIs. The lower risk of reporting bias compared to retrospective studies and the availability of sufficient information regarding cardiovascular background can be considered strengths of the present study. The main findings presented herein are as follows: (i) grade ≥1 CV–irAEs as defined by the ASCO guidelines were more common than previously recognized; (ii) patients with prior acute coronary syndrome and prior heart failure hospitalization were significantly associated with grade ≥1 CV–irAEs, and patients who achieved disease control were also significantly associated with grade ≥1 CV–irAEs; and (iii) patients with preceding grade 1 CV–irAEs had an approximately six times higher risk of subsequent onset of grade ≥2 CV–irAEs compared to patients  without preceding grade 1 CV–irAEs.

First, our prospective observational study showed that grade ≥1 CV–irAEs as defined by the ASCO guidelines were more common than appreciated. Previous studies have reported that the incidence rate of symptomatic ICI–related myocarditis is in the range of 0.06%-3.30%.^3,4^ However, considering the retrospective nature of these studies, and that there were several case reports discussing the occurrence of asymptomatic (subclinical) myocarditis following ICIs,^6-8^ certain CV–irAEs may have remained unidentified or unreported. In the context of underdiagnosis of CV–irAEs, detecting CV–irAEs, including asymptomatic ones, is imperative in the preparation for a potential rechallenge with ICIs. This is especially true considering the results of certain recent studies revealed a 28.8%-55.0% recurrence rate of the same or different grade irAE following rechallenge.^9,10^ Furthermore, one case report showed that rechallenge with ICIs induced a treatment-refractory exacerbation of myocarditis.^8^ Thus, physicians should be sufficiently vigilant to avoid overlooking mild or even asymptomatic CV–irAEs. Another key aspect of the ASCO definition of CV–irAEs is its inclusion of heart failure. Indeed, our study revealed that 5% of the patients receiving ICIs exhibited acute decompensated heart failure or heart failure deterioration in at least one New York Heart Association functional class. Although the present study considers the lack of cardiovascular magnetic  resonance to have caused undiagnosed heart failure due to ICI–related myocarditis, patients with heart failure receiving ICIs should be afforded a high index of clinical suspicion for CV–irAEs considering one case report suggesting that nivolumab promoted dilated cardiomyopathy without inflammatory change.^11^ Of note, CV–irAE was the most frequent adverse event (27%) among the observed irAEs. This result is contradictory to our previous study where skin reaction was the most frequent adverse event,^12^ and this can be explained by the fact that serial cardiac monitoring was not performed in our previous study, and because a dedicated cardiologist was not involved in the diagnosis of CV–irAEs; consequently, CV–irAEs were less recognized in our previous study. Moreover, the underdiagnosis of the skin or gastrointestinal irAEs may have also occurred in the present study because the respective diagnosis was mainly based on the clinical symptoms.

Second, the patients with prior acute coronary syndrome and prior heart failure hospitalization were significantly associated with grade ≥1 CV–irAEs, and patients who achieved disease control were also significantly associated with grade ≥1 CV–irAEs. Regarding the predictors associated with ICI–related myocarditis alone, dual ICI therapy and diabetes were more common in ICI–related myocarditis cases.^3^ Moreover, female sex and age ≥75 years were also associated with an increased risk of ICI–related myocarditis.^13^ However, no reports have indicated the predictors that might affect the incidence of CV–irAEs as defined by the ASCO guidelines, which include not only myocarditis but also pericarditis, arrhythmia, heart failure, and vasculitis.^2^ Thus, our study provides the first evidence that there are several predictors for CV–irAEs that could improve the risk stratification of CV–irAEs. Thus, NSCLC patients with such pre-existing cardiac diseases could be exposed to a higher risk of developing CV–irAEs upon ICI treatment and may benefit more from serial cardiac monitoring. Additionally, our study suggested that ICIs cause more symptomatic cardiovascular toxicity in patients with NSCLC who had a PS ≥ 2 than those who had a PS ≤ 1. Besides, PS at the beginning of treatment retained prognostic significance in patients treated with immunotherapy for NSCLC.^14^ Considering these aspects, clinicians should be more careful about ICI use for patients with NSCLC who had a PS ≥ 2.

Third, the present study showed that patients with preceding grade 1 CV–irAEs had an approximately six times higher risk of subsequent onset of grade ≥2 CV–irAEs compared to patients without preceding grade 1 CV–irAEs. Thus, asymptomatic CV–irAEs (grade 1), which were detected by serial cardiac monitoring, may perhaps indicate the future onset of symptomatic CV–irAEs (grade ≥2). The advantage of serial cardiac monitoring using a combination of BNP, cardiac troponin T, and ECG is that their findings are relatively easy to interpret by physicians not specializing in cardiology considering that differences in the aforementioned factors from baseline are easily recognized after the initiation of ICI treatment. In contrast, serial quantitative troponin I levels were previously investigated for early detection of nivolumab cardiotoxicity in advanced NSCLC.^15^ However, the authors might have underdiagnosed CV–irAEs. The main reason is that they mainly focused on ICI–related myocarditis. Second, complementary measurements of BNP level and ECG were not performed during ICI treatment. Finally, cardiologists were not involved in the diagnosis of ICI–related myocarditis. Although quantitative troponin I measurement would provide more information on the increased risk for major adverse cardiovascular events,^16^ our current study emphasizes the simplicity of interpreting test results (positive or negative results alone for qualitative cardiac troponin T). Thus, our serial cardiac monitoring strategy, including qualitative cardiac troponin T, can become a simple and useful tool for predicting potential grade ≥2 CV–irAEs as long as cardiovascular evaluation before ICI treatment is performed to correctly interpret abnormalities of the variables documented in the serial cardiac monitoring. However, it should be noted that isolated positive biomarker test results without any cardiovascular symptoms should not prompt the discontinuation of ICI treatment, considering that ICIs are key drugs in the treatment of patients with advanced NSCLC, with long-term survival benefits. Even when the patients in our study developed grade ≥2 CV–irAEs, ICI treatment was usually continued because most of the CV–irAEs could be properly managed by dedicated cardiologists without treatment interruption. Only one patient, who developed acute heart failure due to biopsy-proven acute myocarditis, had to discontinue ICI treatment. Therefore, early detection of ICI–related acute myocarditis may be critical in determining whether ICI should be continued. Considering that the sensitivity of cardiac magnetic resonance imaging is not high enough to exclude ICI–related myocarditis,^17^ its negative findings should not eliminate endomyocardial biopsy whenever clinical manifestations involve severe heart failure or life-threatening arrhythmias and are suggestive of ICI–related myocarditis.

### Future Direction

A prospective study of serial cardiac monitoring during dual ICI therapy will be needed because patients who received dual ICI therapy appeared to have more frequent and severe myocarditis than patients who received ICI monotherapy alone.^4^ Additionally, our data revealed that CV–irAE may be a marker of tumor response. The same is true with skin reactions and pneumonitis.^18,19^ However, the reasons why the development of any irAE is associated with tumor response remain unknown. Further research is warranted to uncover the reason for this association.

### Study Limitations

Several limitations of the current study are worth noting. First, considering that our study population was limited to patients with NSCLC, these results cannot be generalized to patients with other types of cancer. Second, quantitative troponin T or I data were not obtained, which could have caused overreporting of CV–irAEs because, with qualitative troponin T data alone, the severity and cause of troponin abnormalities would be more difficult to discern. Third, incidental fluctuations in laboratory test results (ie, the risk of false positives) could not have been completely excluded from grade 1 CV–irAEs, because grade 1 events were defined as laboratory abnormalities without any cardiovascular symptoms. Therefore, overreporting of grade 1 CV–irAEs remains a possibility. Fourth, laboratory data and ECG data were evaluated at every other ICI treatment cycle (every 4-6 weeks), and thus certain events might have been missed, particularly those that occurred within the first six weeks of ICI treatment. This is especially true considering the findings of a previous study showing that the majority of ICI–associated myocarditis cases occurred within the first six weeks of ICI treatment.^20^ Fifth, clinical validation of early detection of CV–irAEs in light of improved overall survival in patients with NSCLC was not clarified. Thus, it remains uncertain whether routine serial testing of BNP, cardiac troponin T, and ECG in unselected patients who underwent ICI treatment should be recommended or not. Sixth, advanced diagnostic examinations, including coronary angiography and endomyocardial biopsy, were performed only in selected patients with grade ≥2 CV–irAEs for further diagnostic workup. As a result, ICI–related myocarditis may have been underdiagnosed. Finally, having no control group was a limitation of our study.

## Conclusions

The current study clearly showed that grade ≥1 CV–irAEs were more common than appreciated. Prior acute coronary syndrome and prior heart failure hospitalization, and achievement of disease control were significantly associated with grade ≥1 CV–irAEs. Furthermore, our results revealed that preceding grade 1 CV–irAEs may confer a significantly higher risk of subsequent onset of grade ≥2 CV–irAEs and that serial cardiac monitoring was feasible for the prediction of future grade ≥2 CV–irAEs. We believe that our study contributes toward increasing awareness for this new clinical entity among all specialists involved in ICI treatment.

## Supplementary Material

oyac056_suppl_Supplementary_Tables

## Data Availability

The participants in this study did not agree to share their data publicly. The de-identified participant data, therefore, will not be shared.
