# Supplementary material for: Incidence, Clinical Characteristics, and Predictors of Cardiovascular Immune-Related Adverse Events Associated with Immune Checkpoint Inhibitors
Source: Oncologist. 2022 Mar 28;27(5):e410–9. doi: 10.1093/oncolo/oyac056 (PMC9074992; doi:10.1093/oncolo/oyac056)
Supplement: oyac056_suppl_Supplementary_Tables [file oyac056_suppl_supplementary_tables.docx]

**Supplementary Table 1. Cardiovascular immune-related adverse events (modified by the authors by referring to the American Society of Clinical Oncology Clinical Practice Guideline 2018).**

| **Cardiovascular immune-related adverse events (CV-irAEs)** | |
| --- | --- |
| Cardiovascular diseases included in CV-irAEs | Myocarditis, pericarditis, arrhythmias, impaired ventricular function with heart failure, and vasculitis. |
| Signs and symptoms of CV-irAEs | Chest pain, arrhythmia, palpitations, peripheral edema, progressive or acute dyspnea, pleural effusion, and fatigue. |
| Grading of CV-irAEs | - Grade 1: Abnormal cardiac biomarker testing, including abnormal electrocardiogram. - Grade 2: Abnormal screening test results with mild symptoms. - Grade 3: Moderately abnormal testing or symptoms with mild activity. - Grade 4: Moderate-to-severe decompensation requiring IV medication or intervention, and life-threatening conditions. |

CV-irAEs, cardiovascular immune-related adverse events.

**Supplementary Table 2. Clinical details of the 129 ICI-treated patients according to the type of ICIs**

| **Type of ICIs** | ***n* (%)** | **Age, median (IQR)** | **Stages II/III/IV, *n* (%)** | **ECOG PS, 0/1/2/3, *n* (%)** | **Prior radiation, *n* (%)** | **Prior chemotherapy, *n* (%)** |
| --- | --- | --- | --- | --- | --- | --- |
| Nivolumab | 47 (36) | 71 (66–77) | 0 (0)/38 (81)/9 (19) | 33 (70)/12 (26)/1 (2)/1 (2) | 12 (26) | 47 (100) |
| Pembrolizumab | 46 (36) | 70 (62–78) | 0 (0)/5 (11)/40 (87)^*^ | 33 (72)/13 (28)/0 (0)/0 (0) | 8 (17) | 2 (4) |
| Atezolizumab | 17 (13) | 72 (65–76) | 0 (0)/3 (18)/14 (82) | 11 (65)/6 (35)/0 (0)/0 (0) | 6 (35) | 17 (100) |
| Durvalumab | 19 (15) | 68 (61–72) | 0 (0)/19 (100)/0 (0) | 18 (95)/1 (5)/0 (0)/0 (0) | 19 (100) | 19 (100) |

^*^Among the patients who received pembrolizumab, one was diagnosed with at least stage II or higher non-small-cell lung cancer and was therefore excluded from the stage classification.

ECOG PS, Eastern Cooperative Oncology Group Performance Status Scale; ICI, immune checkpoint inhibitor; IQR, interquartile range.

**Supplementary Table 3. Univariate associations among baseline clinical, laboratory, and treatment characteristics and grade ≥2 CV-irAEs in patients with NSCLC undergoing ICI treatment based on Fine and Gray competing risk analysis, considering non-cardiovascular death as a competing event**

| **Variables** | | **With CV-irAEs (grade ≥2)**  ***n* = 13** | | **Without CV-irAEs**  ***n* = 94** | | **HR^*^ (95% CI)** | | ***P-*value** | |  |
| --- | --- | --- | --- | --- | --- | --- | --- | --- | --- | --- |
| Age, median (IQR), years | | 65 (71–75) | | 71 (64–77) | | 1.01 (0.96–1.061) | | 0.70 | |  |
| Sex (male), *n* (%) | | 11 (85) | | 72 (77) | | 1.52 (0.34–6.79) | | 0.58 | |  |
| Height, median (IQR), m | | 1.66 (1.60–1.71) | | 1.62 (1.56–1.68) | | 1.03 (0.97–1.09) | | 0.30 | |  |
| Body weight, median (IQR), kg | | 56.0 (47.0–61.0) | | 55.5 (47.0–67.0) | | 0.98 (0.95–1.02) | | 0.29 | |  |
| Body mass index, median (IQR), kg/m^2^ | | 19.4 (18.4–20.1) | | 21.7 (18.4–24.5) | | 0.90 (0.80–1.02) | | 0.10 | |  |
| Diabetes, *n* (%) | | 2 (15) | | 25 (27) | | 0.54 (0.12–2.42) | | 0.42 | |  |
| Hypertension, *n* (%) | | 7 (54) | | 54 (57) | | 0.78 (0.26–2.31) | | 0.65 | |  |
| Active or ex-smoking, *n* (%) | | 12 (92) | | 82 (87) | | 1.63 (0.21–12.80) | | 0.64 | |  |
| Prior stroke, *n* (%) | | 0 (0) | | 4 (4) | | n/a | | n/a | |  |
| Prior ACS, *n* (%) | | 1 (8) | | 2 (2) | | 3.72 (0.47–29.46) | | 0.21 | |  |
|  | |  | |  | |  | |  | |  |
| **(Supplementary Table 3. continued)** | |  | |  | |  | |  | |  |
|  | | **With CV-irAEs (grade ≥2)**  ***n* = 13** | | **Without CV-irAEs**  ***n* = 94** | | **HR^*^ (95% CI)** | | ***P-*value** | |  |
| Prior heart failure hospitalization, *n* (%) | | 1 (8) | | 1 (1) | | 5.40 (0.93–31.33) | | 0.060 | |  |
| Spirometry-defined COPD or emphysema on CT, *n* (%) | | 4 (31) | | 24 (26) | | 1.31 (0.42–4.04) | | 0.64 | |  |
| ECOG PS^†^, *n* (%) | |  | |  | |  | |  | |  |
| 0 | | 10 (77) | | 68 (72) | | 1.03 (0.28–3.80) | | 0.97 | |  |
| 1 | | 2 (15) | | 25 (27) | | 0.60 (0.13–2.73) | | 0.50 | |  |
| ≥2 | | 1 (8) | | 1 (1) | | 51.33 (12.93–203.70) | | < 0.001 | |  |
| Cancer stage^‡^, *n* (%) | |  | |  | |  | |  | |  |
| III | | 4 (31) | | 28 (30) | | 1.20 (0.37–3.90) | | 0.76 | |  |
| IV | | 9 (69) | | 65 (69) | | 0.87 (0.27–2.83) | | 0.82 | |  |
| Pathological subtype, *n* (%) | |  | |  | |  | |  | |  |
| SCC | | 8 (61) | | 43 (46) | | 0.58 (0.19–1.75) | | 0.33 | |  |
| Nonsquamous NSCLC | | 5 (39) | | 51 (54) | |  |  |  |  |  |
|  | |  | |  | |  | |  | |  |
| **(Supplementary Table 3. continued)** | |  | |  | |  | |  | |  |
|  | | **With CV-irAEs (grade ≥2)**  ***n* = 13** | | **Without CV-irAEs**  ***n* = 94** | | **HR^*^ (95% CI)** | | ***P-*value** | |  |
| Prior use of anthracyclines, *n* (%) | | 0 (0) | | 0 (0) | | n/a | | n/a | |  |
| Prior use of VEGF inhibitors^§^, *n* (%) | | 2 (15) | | 10 (11) | | 1.24 (0.25– 6.11) | | 0.79 | |  |
| Prior radiation, *n* (%) | | 4 (31) | | 38 (40) | | 0.70 (0.22– 2.25) | | 0.55 | |  |
| PD-L1 expression, *n* (%) | |  | |  | |  | |  | |  |
| TPS ≥50% | | 2 (15) | | 17 (20) | | 0.73 (0.19–2.84) | | 0.65 | |  |
| Nivolumab, *n* (%) | | 8 (61) | | 31 (33) | | 2.62 (0.86–8.00) | | 0.090 | |  |
| Pembrolizumab, *n* (%) | | 4 (31) | | 32 (34) | | 0.94 (0.30–3.00) | | 0.92 | |  |
| Atezolizumab, *n* (%) | | 1 (8) | | 13 (14) | | 0.49 (0.06–4.07) | | 0.51 | |  |
| Durvalumab, *n* (%) | | 0 (0) | | 18 (19) | | n/a | | n/a | |  |
| Best response, *n* (%) | |  | |  | |  | |  | |  |
| Complete response | | 0 (0) | | 1 (1) | | n/a | | n/a | |  |
| Partial response | | 3 (23) | | 26 (28) | | 0.93 (0.26–3.30) | | 0.91 | |  |
| Stable disease | | 6 (46) | | 20 (21) | | 3.07 (1.07–8.79) | | 0.036 | |  |
|  | |  | |  | |  | |  | |  |
| **(Supplementary Table 3. continued)** | |  | |  | |  | |  | |  |
|  | | **With CV-irAEs (grade ≥2)**  ***n* = 13** | | **Without CV-irAEs**  ***n* = 94** | | **HR^*^ (95% CI)** | | ***P-*value** | |  |
| Progressive disease | | 4 (31) | | 47 (50) | | 0.39 (0.11–1.34) | | 0.14 | |  |
| Objective response^?^ | | 3 (23) | | 27 (29) | | 0.89 (0.25–3.16) | | 0.85 | |  |
| Disease control^#^ | | 9 (69) | | 47 (50) | | 2.54 (0.75–8.62) | | 0.14 | |  |
| Pre-ICI treatment white blood cells (×10^3^), median (IQR), per µL | | 5.8 (5.2– 6.8) | | 6.2 (4.3–7.5) | | 1.00 (n/a) | | 0.70 | |  |
| Pre-ICI treatment hemoglobin, median (IQR), g/dL | | 12.7 (12.0–13.8) | | 12.1 (10.8–13.0) | | 1.22 (0.93–1.61) | | 0.15 | |  |
| Pre-ICI treatment eGFR, median (IQR), mL/min/1.73 m^2^ | | 73.7 (67.0–79.9) | | 75.9 (60.7–87.4) | | 1.01 (0.99–1.03) | | 0.43 | |  |
| Pre-ICI treatment BNP, median (IQR), pg/mL | | 41.0 (24.7–90.3) | | 26.7 (13.5–62.6) | | 1.00 (n/a) | | 0.98 | |  |
| Clinically relevant BNP elevation (≥200 pg/mL) at start of ICI treatment^**^, *n* (%) | | 0 (0) | | 3 (3) | | n/a | | n/a | |  |
|  | |  | |  | |  | |  | |  |
|  | |  | |  | |  | |  | |  |
|  | |  | |  | |  | |  | |  |
| **(Supplementary Table 3. continued)** | |  | |  | |  | |  | |  |
|  | | **With CV-irAEs (grade ≥2)**  ***n* = 13** | | **Without CV-irAEs**  ***n* = 94** | | **HR^*^ (95% CI)** | | ***P-*value** | |  |
| Pre-ICI treatment echocardiogram | |  | |  | |  | |  | |  |
| LVEF, median (IQR), % | | 75 (63–78) | | 70 (66–75) | | 1.03 (0.95–1.11) | | 0.53 | |  |
| LVEd, median (IQR), mm | | 43(43–44) | | 44 (41–48) | | 0.97 (0.90–1.05) | | 0.43 | |  |
| LVEs, median (IQR), mm | | 24 (22–28) | | 27 (24–30) | | 0.93 (0.83–1.05) | | 0.25 | |  |
| Clinically relevant LVEF dysfunction (<50%) at the start of ICI treatment^**^, *n* (%) | | 0 (0) | | 2 (2) | | n/a | | n/a | |  |
| Cardiac troponin T prior to ICI use, *n* (%) | |  | |  | |  | |  | |  |
| Negative | | 0 (0) | | 2 (2) | | n/a | | n/a | |  |
| Pre-ICI treatment ECG | |  | |  | |  | |  | |  |
| Persistent/paroxysmal atrial fibrillation, *n* (%) | | 0 (0) | | 15 (16) | | n/a | | n/a | |  |
| ST-T changes, *n* (%) | | 0 (0) | | 2 (2) | | n/a | | n/a | |  |
| Negative T-wave, *n* (%) | | 0 (0) | | 1 (1) | | n/a | | n/a | |  |
| Heart rate at rest, median (IQR), per min | | 75 (67–86) | | 80 (68–87) | | 0.99 (0.96–1.02) | | 0.52 | |  |
|  | |  | |  | |  | |  | |  |
| **(Supplementary Table 3. continued)** | |  | |  | |  | |  | |  |
|  | | **With CV-irAEs (grade ≥2)**  ***n* = 13** | | **Without CV-irAEs**  ***n* = 94** | | **HR^*^ (95% CI)** | | ***P-*value** | |  |
| QRS width, median (IQR), ms | | 90 (84–120) | | 90 (80–100) | | 1.02 (0.99–1.04) | | 0.20 | |  |
| Other organ systems affected by irAEs, *n* (%) | |  | |  | |  | |  | | |
| Skin reaction | | 1 (8) | | 15 (16) | | 0.49 (0.06–3.84) | | 0.50 | | |
| Myositis/peripheral neuropathy | | 0 (0) | | 2 (2) | | n/a | | n/a | | |
| Hypothyroidism/hyperthyroidism | | 1 (8) | | 8 (9) | | 0.97 (0.13–7.41) | | 0.98 | | |
| Pneumonitis | | 3 (23) | | 17 (18) | | 1.50 (0.43–5.29) | | 0.52 | | |
| Hepatitis | | 0 (0) | | 8 (9) | | n/a | | n/a | | |
| Diarrhea | | 1 (8) | | 1 (1) | | 4.29 (0.81–22.77) | | 0.087 | | |
| Nephritis | | 0 (0) | | 1 (1) | | n/a | | n/a | | |
| Adrenal insufficiency | | 0 (0) | | 4 (4) | | n/a | | n/a | | |
| Any irAEs other than CV-irAEs | | 5 (39) | | 43 (46) | | 0.75 (0.25–2.23) | | 0.60 | | |

^*^ 'Without CV-irAEs' was considered a reference category.

^†^ Scores range from 0 to 4, with higher numbers indicating greater disability.

^‡^ Among the 129 patients analyzed, one was diagnosed as at least stage II or higher NSCLC and was therefore excluded from either stage III or stage IV.

^§^ VEGF inhibitors included bevacizumab and ramucirumab.

^?^ Achieving complete or partial response.

^#^ Achieving complete response, partial response, or stable disease.

^**^ The term, “clinically relevant”, was defined as either BNP elevation (≥200 pg/mL) or LVEF dysfunction (<50%) originating from symptomatic (New York Heart Association class II or greater) chronic heart failure at the beginning of ICI treatment.

ACEI, angiotensin-converting enzyme inhibitor; ACS, acute coronary syndrome; ARB, angiotensin II receptor blocker; BNP, B-type natriuretic peptide; CI, confidence interval; CT, computed tomography; COPD, chronic obstructive pulmonary disease; CV-irAEs, cardiovascular immune-related adverse events; ECOG PS, Eastern Cooperative Oncology Group Performance Status Scale; ECG, electrocardiogram; eGFR, estimated glomerular filtration rate; HR, hazard ratio; ICI, immune checkpoint inhibitor; IQR, interquartile range; LVEd, left ventricular end-diastolic dimension; LVEF, left ventricular ejection fraction; LVEs, left ventricular end-systolic dimension; n/a, not applicable; NSCLC, non-small-cell lung cancer; PD-L1, programmed death-ligand 1; SCC, squamous cell carcinoma; TPS, tumor proportion score; VEGF, vascular endothelial growth factor.

**Supplementary Table 4.** **Univariate and multivariate Fine and Gray regression models for grade ≥2 CV-irAEs in patients with NSCLC undergoing ICI treatment, considering non-cardiovascular death as a competing event**

| **Variables** | **Univariate** | |  | **Multivariate^*^** | |
| --- | --- | --- | --- | --- | --- |
|  | **HR (95% CI)** | ***P-*value** |  | **AHR (95% CI)** | ***P-*value** |
| ECOG PS ≥2 | 51.33 (12.93–203.70) | < 0.001 |  | 84.62 (18.43–388.40) | < 0.001 |
| Achievement of stable disease | 3.07 (1.07–8.79) | 0.036 |  | 3.63 (1.22–10.77) | 0.020 |

^*^Adjusted for ECOG PS ≥2 and achievement of stable disease.

AHR, adjusted hazard ratio; CI, confidence interval; CV-irAEs, cardiovascular immune-related adverse events; ECOG PS, Eastern Cooperative Oncology Group Performance Status Scale; HR, hazard ratio; ICI, immune checkpoint inhibitor; NSCLC, non-small-cell lung cancer.
